# Brain Functional Connectivity Correlates of Response in the 7.5% CO_2_ Inhalational Model of Generalized Anxiety Disorder: A Pilot Study

**DOI:** 10.1093/ijnp/pyaa019

**Published:** 2020-03-14

**Authors:** Nathan T M Huneke, M John Broulidakis, Angela Darekar, David S Baldwin, Matthew Garner

**Affiliations:** 1 Clinical and Experimental Sciences, Faculty of Medicine, University of Southampton, Southampton, United Kingdom; 2 Department of Medical Physics, University Hospital Southampton NHS Foundation Trust, Southampton, United Kingdom; 3 Southern Health NHS Foundation Trust, Southampton, United Kingdom; 4 University Department of Psychiatry and Mental Health, University of Cape Town, Cape Town, South Africa; 5 Academic Unit of Psychology, Faculty of Environmental and Life Sciences, University of Southampton, Southampton, United Kingdom

**Keywords:** Anxiety, CO_2_ challenge, experimental medicine, fMRI, functional connectivity

## Abstract

**Background:**

The 7.5% CO_2_ inhalational model can be used to explore potential treatments for generalized anxiety disorder. However, it is unknown how inter-individual variability in the functional architecture of negative affective valence systems might relate to anxiogenic response in this model.

**Methods:**

A total of 13 healthy volunteers underwent functional magnetic resonance imaging during a passive emotional face perception task. We explored task-evoked functional connectivity in the potential threat system through generalized psychophysiological interaction analysis. Within 7 days, these participants underwent prolonged 7.5% CO_2_ inhalation, and results from the generalized psychophysiological interaction analysis were correlated with CO_2_ outcome measures.

**Results:**

Functional connectivity between ventromedial prefrontal cortex and right amygdala positively correlated with heart rate and subjective anxiety, while connectivity between midcingulate cortex and left amygdala negatively correlated with anxiety during CO_2_ challenge.

**Conclusions:**

Response to CO_2_ challenge correlated with task-evoked functional connectivity in the potential threat system. Further studies should assess whether this translates into clinical populations.

## Introduction

Experimental medicine models in healthy volunteers can be a cost-effective and timely approach to explore potential novel treatments for psychiatric disorders. An example is the 7.5% CO_2_ inhalational model of generalized anxiety disorder (GAD), in which healthy volunteers inhale air “enriched” with 7.5% CO_2_ (“CO_2_ challenge”). This model mimics the subjective, autonomic, and neuropsychological features of GAD (Bailey et al., 2007; Garner et al., 2011). Anxiety induced in this model is responsive to standard pharmacological and psychological treatment (Bailey et al., 2007; Ainsworth et al., 2015). Therefore, this model provides an approach for testing potential treatments for GAD at a “proof of concept” stage before embarking on time-consuming and costly phase II/III clinical trials.

However, there is variation in how healthy volunteers respond to CO_2_ challenge. Volunteers with high trait anxiety and anxiety sensitivity experience increased subjective and physiological responses to CO_2_ challenge compared with those with lower scores (Olatunji et al., 2009; Fluharty et al., 2016). Both increased anxiety sensitivity and 7.5% CO_2_ inhalation increase selective attention biases to threatening stimuli (Keogh et al., 2001; Garner et al., 2011). This suggests that the function of “negative affective valence systems” (defined by NIMH research domain criteria https://www.nimh.nih.gov/research/research-funded-by-nimh/rdoc/index.shtml), particularly in the constructs of acute and potential threat, might be important. Supporting this, 7% CO_2_ inhalation in panic disorder patients causes increased brainstem activity, a region within the acute threat system (Goossens et al., 2014).

A key anatomical node in the negative affective valence system is the amygdala. Amygdala hyperactivity during negative emotion processing is commonly reported in patients with GAD (Mochcovitch et al., 2014) and exhibits abnormal functional connectivity with emotion-processing regions at rest and during fearful face perception (Prater et al., 2013; Rabany et al., 2017). Similarly, functional connectivity of the amygdala also relates to anxiety measures in healthy volunteers. For example, trait anxiety correlates negatively with connectivity between the midcingulate cortex (MCC) and the left amygdala when viewing negative pictures (Kienast et al., 2008). It is therefore possible that inter-individual differences in the functional architecture of negative affective valence systems might be a biomarker of prospective subjective and physiological response to CO_2_ challenge.

We carried out a pilot study to explore how functional connectivity within negative affective valence networks is associated with response to CO_2_ challenge in healthy volunteers. To probe functional connectivity of these networks under different conditions, we used a validated emotional face perception task known to activate the amygdala (Grosbras and Paus, 2006; Schneider et al., 2011). Through generalized psychophysiological interaction (gPPI) analysis, we identified task-evoked changes in functional connectivity and correlated these findings with CO_2_ outcome measures. Our aim was to identify whether there were any signals of interest to inform future studies.

## METHODS

### Ethics Statement

This study was reviewed and approved by the Ethics and Research Governance Office at the University of Southampton (reference: 27440). All participants provided written, informed consent.

### Participants

We recruited 13 healthy volunteers (mean age 23.38 ± 4.27 years, 8 females) from the community. Exclusion criteria included current or lifetime history of psychiatric illness (as assessed by the Mini International Neuropsychiatric Interview for DSM-IV-MINI) (Sheehan et al., 1998); chronic physical illness; alcohol or drug dependence; use of medication, alcohol, or illicit drugs in the previous 8 weeks; regular smokers; any contraindication to MRI scanning; and body mass index <18 or >28 kg/m^2^.

### Study Design

#### Trait Anxiety Measures

**—**Following screening, eligible participants completed the following baseline measures of trait anxiety: State-Trait Anxiety Inventory (Spielberger et al., 1983); Anxiety Sensitivity Index (Peterson and Reiss, 1992); and a modified version of the GAD-7 (Spitzer et al., 2006), where each question was represented by a visual analogue scale ranging from “not at all” to “nearly every day.”

#### MRI Scanning Session

**—**Participants attended University Hospital Southampton (Southampton, UK) for a functional magnetic resonance imaging (fMRI) scan. During the scan, participants completed a validated passive emotional face perception task (described further in supplementary material) (Grosbras and Paus, 2006). In brief, participants passively viewed 18-second video clips of actors making happy, angry, or neutral facial expressions. These clips were interspersed with an 18-second control condition of expanding and contracting black and white concentric circles. Participants viewed 4 blocks of each emotional expression and 12 blocks of the control condition (total 24 blocks). This task has been shown to reliably activate the amygdala (Schneider et al., 2011) and would therefore be a good probe of negative affective valence network function.

#### CO_2_ Challenge

**—**Within 7 days of the MRI scan, participants completed two 20-minute inhalations of normal air and air enriched with 7.5% CO_2_ (21% O_2_, balance N_2_) administered through an oronasal face mask under single-blind conditions. We measured heart rate, blood pressure, subjective mood (Positive and Negative Affect Schedule) (Watson, 1988) and anxiety at pretest baseline and immediately following each 20-minute inhalation of air and 7.5% CO_2_. Subjective state anxiety was measured with a modified version of the GAD-7, where each question was represented by a visual analogue scale ranging from “not at all” to “all of the time.”

### Image Acquisition

Images were acquired using a 20-channel head coil on a 3T Siemens Skyra MRI scanner (Siemens Healthineers Limited). T1-weighted (MP-RAGE) anatomical images were acquired for registration purposes. fMRI were obtained with a T2*-weighted single-shot gradient echo, echo planar imaging sequence (see supplementary Materials). The session consisted of 178 volumes synchronized with the onset of the experimental task.

### Image Preprocessing

We used FMRI Expert Analysis Tool version 5.0.8 for image preprocessing. Preprocessing steps included slice time correction, motion correction, brain extraction, spatial smoothing, and application of a high pass filter with a 160-second cutoff. Functional images were registered to high resolution structural images (T1 MP-RAGE) and then to standard space images (T1-weighted Montreal Neurological Institute template) for group-level analyses. Further detail can be found in the supplementary Materials.

### Statistical Analyses

#### Analysis of CO_2_ Challenge

**—**We assessed response to CO_2_ challenge through ANOVA with repeated measures for each outcome measure with time as the within-subject factor (pre-test baseline, post-air, and post-CO_2_) using the SPSS software package (IBM SPSS Statistics for Windows version 24.0; IBM Corp., Armonk, NY). We used Greenhouse-Geisser correction where sphericity assumptions were violated.

#### Functional MRI Analyses

**—**We initially analyzed task-evoked activation through voxelwise whole-brain analysis in the contrast all faces >control. We applied a general linear model to the fMRI time-series per task condition convolved with a canonical hemodynamic response function at the individual level. Group analysis was carried out with a mixed effects statistical model with family wise error correction at a significance threshold of *P* = .05 (see supplementary Materials).

We explored task-modulated functional connectivity through gPPI analysis using the CONN toolbox (www.nitrc.org/projects/conn, RRID:SCR_009550). Connectivity analysis can confer information regarding functional integration of brain regions that is not observable by simple change in BOLD signal (Rao et al., 2008). We restricted the analysis to ten 6-mm spherical regions of interest defined from significant activity seen in a separate sample of 100 young adults who were scanned while completing the same emotional face perception task for another study in the department (see supplementary Materials). For each seed region of interest, bivariate regression matrices were calculated, yielding standardized regression coefficients at the group level. CO_2_ challenge outcome measures were included as group-level covariates of interest to assess the relationship between task-evoked functional connectivity and 7.5% CO_2_-induced anxiety. We explored the contrasts all faces >control to assess for effects of task and angry >happy to assess emotional valence effects. Significance was defined as *q* < 0.05 (2-tailed, false discovery rate correction at the level of the entire analysis). As this was a pilot study, we also performed exploratory analyses with an uncorrected threshold of *P* < .01.

## Results

### Trait Anxiety Measures

At baseline, participants’ mean (±SD) trait anxiety measures were: State-Trait Anxiety Inventory-trait, 31.31 (±9.79); Anxiety Sensitivity Index, 7.77 (±4.6); GAD-7, 1.44 (±1.07). Our sample showed low trait anxiety and low anxiety sensitivity.

### CO_2_ Challenge Results

CO_2_ challenge significantly increased subjective anxiety (F_(1,15)_ = 12.89, *P* = .002), negative affect (F_(1,13)_ = 7.45, *P* = .015), systolic blood pressure (F_(2,22) _= 6.57, *P* = .007), and heart rate (F_(2,19)_ = 12.26, *P* = .001) (supplementary Table 2).

### fMRI Analysis

#### Whole-Brain Voxelwise Analysis

**Figure 1.**
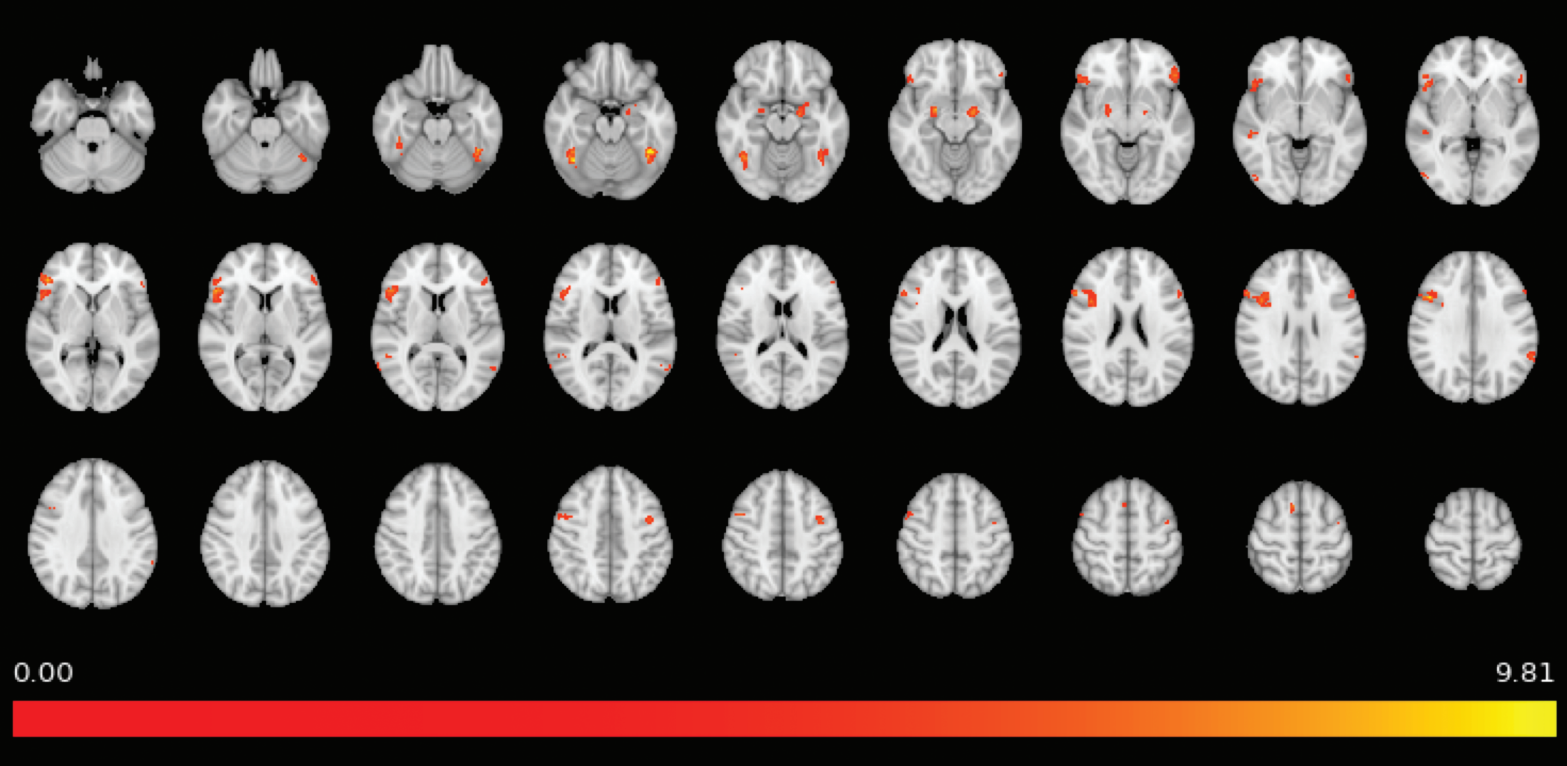
Significant activation seen on whole-brain analysis of the contrast all faces >control in the passive emotional face perception task; all Zs are >5.91, all voxelwise corrected *P* < .0005. Color bar represents Z value.

**—**We initially analyzed activity in the contrast All faces >control to ensure that participants responded to the task as expected. As expected, areas including right middle frontal gyrus, bilateral amygdala, left orbitofrontal, and bilateral fusiform cortex were significantly activated (all *Z* > 5.91, all corrected *P* < .0005) (Figure 1; supplementary Table 3).

#### gPPI Analys**is**

**Figure 2.**
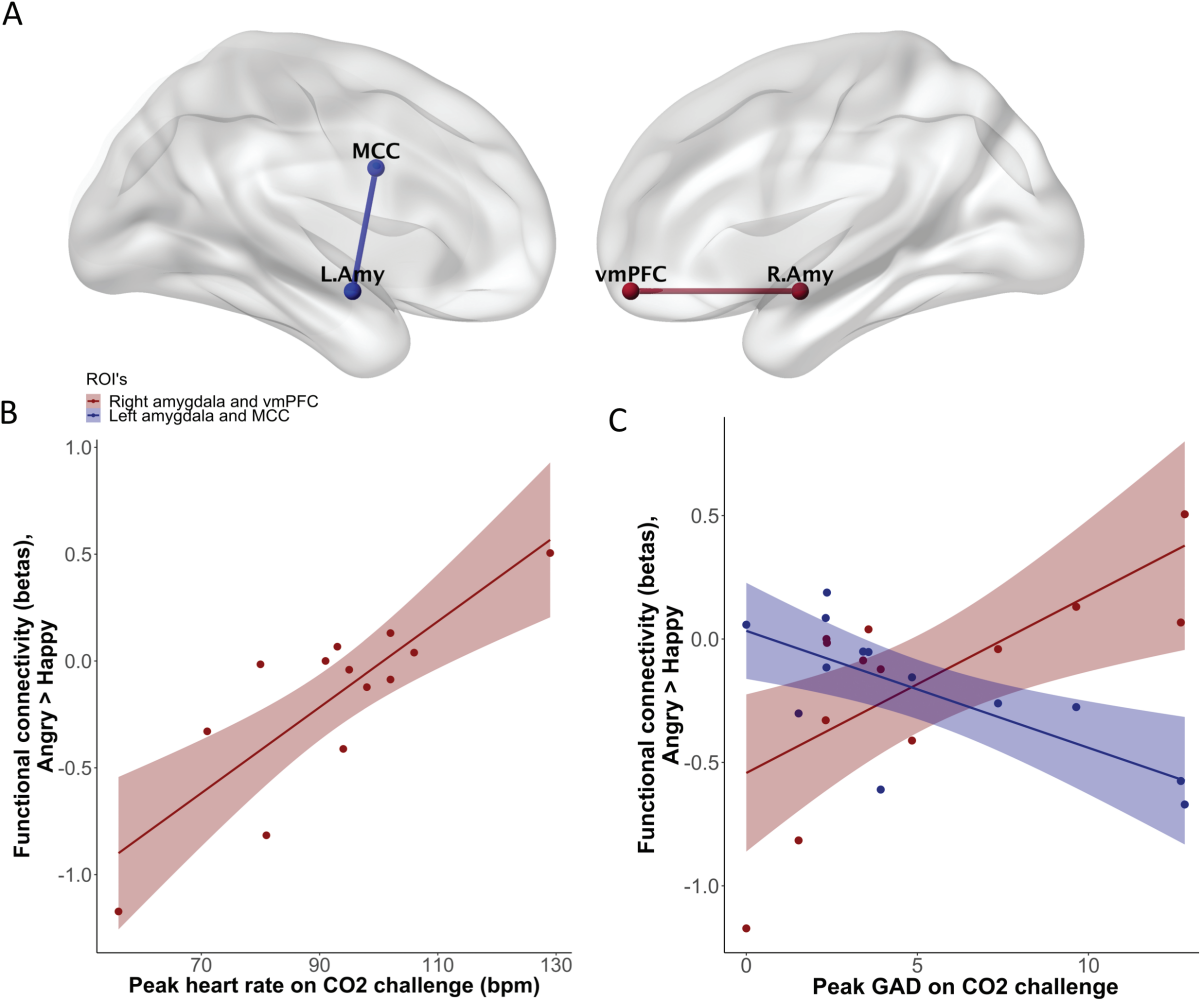
(A) Task-evoked functional connectivity (contrast angry >happy) between right amygdala and ventromedial prefrontal cortex (vmPFC) and between left amygdala and midcingulate cortex (MCC) correlated with CO_2_ outcome measures. These connectivity results were visualized with the BrainNet Viewer (http://www.nitrc.org/projects/bnv/). B and C are scatter plots showing the association of functional connectivity in these regions of interest with heart rate and peak subjective anxiety during CO_2_ challenge. bpm, beats per minute; GAD, generalized anxiety disorder screener.

**—**We did not find a main effect of task on gPPI connectivity estimates. We next included CO_2_ outcome measures as covariates of interest. Heart rate following CO_2_ challenge was positively correlated with functional connectivity between the ventromedial prefrontal cortex (vmPFC) and right amygdala in the contrast angry >happy (r = 0.831, t_(11)_ = 4.96, *q* = 0.0039) (Figure 2). There were no other significant relationships between baseline trait anxiety measures, CO_2_ outcome measures, and task-evoked functional connectivity when correcting for multiple comparisons.

We also carried out exploratory analyses with a more liberal threshold of uncorrected *P* < .01. With this threshold we found additional results, again in the contrast angry >happy. GAD-7 scores were positively correlated with functional connectivity between the vmPFC and right amygdala (r = 0.700, t_(11)_ = 3.25, *P* = .0078), and negatively correlated with connectivity between the MCC and left amygdala (r = −0.726, t_(11)_ = −3.50, *P* = .0050). For interest, additional correlations with moderate-to-large effect size are in supplementary Table 4.

## Discussion

We explored how inter-individual variability in the functional architecture of negative affective valence networks is associated with response to the CO_2_ challenge. Consistent with previous literature, inhalation of 7.5% CO_2_ significantly increased subjective and autonomic anxiety measures (Bailey et al., 2007; Garner et al., 2011). We also found a significant relationship between anxiety during CO_2_ challenge and functional connectivity when viewing angry compared with happy faces. Increased connectivity between the vmPFC and the amygdala was positively correlated with increased heart rate and with subjective anxiety, while subjective anxiety was negatively correlated with connectivity between the MCC and the amygdala.

Increased vmPFC-right amygdala connectivity when viewing angry compared with happy faces was significantly correlated with heart rate, and at a trend level with subjective anxiety, during CO_2_ challenge. Previous studies have shown a relationship between vmPFC activity, sympathetic tone, and anxiety. Regional cerebral blood flow in the vmPFC during passive anticipation of shock correlates with both heart rate and subjective anxiety (Simpson et al., 2001), and patients with vmPFC damage exhibit reduced skin conductance responses to negative images (Damasio et al., 1990). Further, evidence suggests that anterior vmPFC and amygdala co-activation occurs during processing of potential threat. For example, when healthy volunteers attempted to “escape” from a virtual predator, potential threat was associated with activity in the vmPFC and right basolateral amygdala, while during acute threat activity shifted to a midbrain network including central amygdala and periacqueductal grey matter (Mobbs et al., 2007). Also, adults with psychopathy exhibit reduced fear-potentiated startle to environmental threats, reduced amygdala responses to aversive emotional stimuli, and reduced functional connectivity between vmPFC and the amygdala (Blair, 2013). It is possible that increased connectivity between vmPFC and the amygdala relates to a lower threshold to appraise aversive stimuli as threatening. This could explain why participants who show this pattern of activity when viewing aversive stimuli respond more convincingly to CO_2_ challenge.

GAD-7 scores were negatively associated at a trend level with functional connectivity between the MCC and the left amygdala when viewing angry compared with happy faces. These results are similar to the findings of a previous study of healthy adult men: functional connectivity between the left MCC and the left amygdala when viewing negative compared with neutral faces was negatively correlated with trait anxiety (Kienast et al., 2008). The MCC appears to play a complex role in selecting appropriate actions based on incoming negatively valenced information (Vogt, 2014). It is possible that reduced functional connectivity between this region and the amygdala suggests reduced ability to engage appropriate control mechanisms to manage aversive emotional states.

Our methods had some limitations. First, this was an exploratory pilot study with a small sample size and a relatively short behavioral task during the scan. Not only does this increase the chance of type I error, we also did not have statistical power to carry out seed to voxel or voxelwise gPPI analyses. Second, there is potentially an order confound, as all participants completed the fMRI scan prior to CO_2_ challenge. Third, given previous findings that 7% CO_2_ inhalation causes increased brainstem activity in those with panic disorder (Goossens et al., 2014), it would have been interesting to explore interactions between brainstem and higher cortical centers. The methods used in this study were not appropriate to make meaningful inferences about brainstem activity, but this should be explored in future studies. Fourth, we did not measure subjective mood or anxiety during the scanning session; thus, it is unknown whether participants experienced similar emotions during scanning and CO_2_ challenge. However, the regions activated by the faces task were consistent with the literature (Grosbras and Paus, 2006; Schneider et al., 2011), including the amygdala, which suggests the task indeed recruited negative valence systems. Nevertheless, our results must be interpreted with caution due to these limitations.

The purpose of this study was to explore the association between the functional architecture of negative affective valence networks and response to CO_2_ challenge in healthy volunteers. Our preliminary findings suggest that increased connectivity between vmPFC and right amygdala, and reduced connectivity between MCC and left amygdala, when viewing aversive stimuli is associated with a greater anxiogenic response. A hypothesis that could explain these findings is that this activity pattern occurs in participants with a lower threshold to assess aversive stimuli as threatening and who have reduced ability to then manage this emotional response. Further studies should be undertaken to test this hypothesis and whether this translates into clinical populations.

## Supplementary Material

pyaa019_suppl_Supplementary_material

## Interest Statement

NTMH conducted this work as a National Institute of Health Research (NIHR) Academic Clinical Fellow. NTMH is now a Medical Research Council (MRC) clinical research fellow. The NIHR and MRC had no role in the design of the study, collection, and analysis of the data or the decision to publish. Dr Broulidakis, Dr Darekar, Prof Baldwin, and Prof Garner declare no potential conflict of interest.
