# Supplementary material for: Brain Functional Connectivity Correlates of Response in the 7.5% CO2 Inhalational Model of Generalized Anxiety Disorder: A Pilot Study
Source: Int J Neuropsychopharmacol. 2020 Mar 14;23(4):268–73. doi: 10.1093/ijnp/pyaa019 (PMC7177158; doi:10.1093/ijnp/pyaa019)
Supplement: pyaa019_suppl_Supplementary_material [file pyaa019_suppl_supplementary_material.zip › Supplementary-material.rtf]

Supplementary Material
1.	Supplementary methods
1.1.	Passive emotional face perception task
Participants passively viewed short black and white video clips of actors making emotional facial expressions. The actors' faces always started from a neutral expression, and then either turned angry, or happy, or the actor made a neutral movement with no emotional content. These stimuli were arranged into 18-second blocks of faces portraying the same emotion. These blocks were interspersed with an 18-second control condition of expanding and contracting black and white concentric circles. Altogether, participants viewed 4 blocks of happy faces, 4 blocks of angry faces, 4 blocks of neutral faces, and 12 blocks of the control condition (total 24 blocks, approximately 7.2 minutes). 
1.2.	Image Acquisition
T1-weighted (MP-RAGE) images were acquired for registration purposes (voxels 1mm3; TR 2200ms; TE 2.45ms; flip angle 8 degrees; 176 sagittal slices). Functional MR images were obtained with a T2*-weighted single-shot gradient echo, echo planar imaging sequence (voxels 2.5mm3; TR 2500ms; TE 30ms; parallel imaging method: GRAPPA with acceleration factor 4; flip angle 90 degrees; FOV 235mm x 235mm; 45 slices in an oblique orientation, and interleaved acquisition 2.5mm slice thickness no gap).
1.3.	MR image pre-processing
We used FEAT (FMRI Expert Analysis Tool) version 5.0.8, part of FSL (FMRIB's Software Library, www.fmrib.ox.ac.uk/fsl) for image preprocessing. Preprocessing steps included slice time correction, motion correction using MCFLIRT (Jenkinson et al., 2002), brain extraction, spatial smoothing with a gaussian filter with a full width at half maximum kernel of 5mm, and application of a high pass filter with a 160s cutoff. None of our participants had movement greater than 2mm translation or 2 degrees rotation. We carried out linear registration of functional images to high resolution structural images (T1 MP-RAGE) and then to standard space images (T1-weighted Montreal Neurological Institute template) with 12 degrees of freedom, using FLIRT (Jenkinson and Smith, 2001; Jenkinson et al., 2002). Registration from high resolution structural to standard space was then further refined with FNIRT nonlinear registration (Andersson et al., 2007a; Andersson et al., 2007b).
1.4.	Functional MRI analyses
We first performed a whole brain voxelwise analysis of activity in the contrast All Faces > Control to check whether participants responded to the emotional face perception task as expected. At the individual level, we applied a general linear model to the fMRI timeseries for each task condition convolved with a single-gamma canonical haemodynamic response function. Higher level analysis was carried out using a mixed effects statistical model (FLAME stage 1 and stage 2 (Beckmann et al., 2003; Woolrich et al., 2004; Woolrich, 2008)). We applied voxel-wise control of family-wise error with a (corrected) significance threshold of p = 0.05.
We explored task-modulated functional connectivity through generalized psychophysiological interaction (gPPI) analysis using the CONN toolbox (www.nitrc.org/projects/conn, RRID:SCR_009550). In brief, this analysis involves calculating a separate multiple regression model for target regions of interest, each including psychological predictors (all task effects convolved with a canonical haemodynamic response function) and physiological predictors (average BOLD timeseries from seed region), and the interaction of these (their product). We carried out generalized PPI as this has been shown to better capture task effects compared with standard PPI methods (McLaren et al., 2012). Due to our small sample size we restricted the analysis to 10 regions of interest (ROI) to improve signal to noise ratio. We defined these from a sample of 100 young adults who were scanned while undergoing the same passive emotional face perception task for another study carried out in the department. Spheres with radius of 6mm were created from peak coordinates in significant clusters seen in the contrast All Faces > Control in this sample (Z > 2.3, corrected p < 0.05) (see Supplementary Table S1). 
For each seed ROI, bivariate regression matrices were calculated, yielding standardised regression coefficients at the group level. We included motion parameters as a confound regressor at the lower level. CO2 challenge outcome measures were included as higher-level covariates of interest to assess the relationship between task-evoked functional connectivity and response to 7.5% CO2 inhalation. We explored the contrasts All faces > control to assess for effects of task and angry > happy to assess the effects of emotional valence. We report results at a threshold of q < 0.05 (2-tailed) with false discovery rate (FDR) correction at the level of the entire analysis (i.e. controlling for each ROI to target pair simultaneously). As this was a pilot study, we also performed exploratory analyses with an uncorrected significance threshold of p < 0.01 (2-tailed).
2.	Supplementary References
Andersson J, Jenkinson M, Smith S (2007a) Non-linear optimisation. FMRIB Analysis Group Technical Reports. TR07JA1. In: Oxford: University of Oxford.
Andersson JL, Jenkinson M, Smith S (2007b) Non-linear registration aka Spatial normalisation FMRIB Technial Report TR07JA2. FMRIB Analysis Group of the University of Oxford.
Beckmann CF, Jenkinson M, Smith SM (2003) General multilevel linear modeling for group analysis in FMRI. Neuroimage 20:1052-1063.
Jenkinson M, Smith S (2001) A global optimisation method for robust affine registration of brain images. Med Image Anal 5:143-156.
Jenkinson M, Bannister P, Brady M, Smith S (2002) Improved optimization for the robust and accurate linear registration and motion correction of brain images. Neuroimage 17:825-841.
McLaren DG, Ries ML, Xu G, Johnson SC (2012) A generalized form of context-dependent psychophysiological interactions (gPPI): a comparison to standard approaches. Neuroimage 61:1277-1286.
Schneider S, Peters J, Bromberg U, Brassen S, Menz MM, Miedl SF, Loth E, Banaschewski T, Barbot A, Barker G (2011) Boys do it the right way: sex-dependent amygdala lateralization during face processing in adolescents. Neuroimage 56:1847-1853.
Woolrich M (2008) Robust group analysis using outlier inference. Neuroimage 41:286-301.
Woolrich MW, Behrens TE, Beckmann CF, Jenkinson M, Smith SM (2004) Multilevel linear modelling for FMRI group analysis using Bayesian inference. Neuroimage 21:1732-1747.


3.	Supplementary Tables
Supplementary Table S1: Regions of interest used in the generalized psychophysiological interaction analysis. We defined these from significant activity seen in a separate sample of 100 young adults who were scanned while completing the same emotional face perception task (Z > 2.3, corrected p < 0.05).
		MNI coordinates	
Anatomical Region		x	y	z	
Right					
	Amygdala	20	-4	-14	
	SMA	4	10	60	
	Angular gyrus	34	-56	40	
	Thalamus	12	-14	8	
	vmPFC	4	54	-14	
Left					
	Amygdala	-20	-4	-14	
	Caudate	-14	-6	18	
	Superior temporal gyrus	-50	4	-18	
	MCC	-6	4	28	
	Supramarginal gyrus	-50	-48	10	
Abbreviations: MNI, Montreal Neurological Institute; SMA, supplementary motor area; vmPFC, ventromedial prefrontal cortex; MCC, midcingulate cortex.	


Supplementary Table S2: Summary of CO2 challenge results analysed through repeated measures ANOVAs
Outcome measure	Pre Air	Post Air	Post CO2	ANOVA	
GAD-7	1.43 ± 0.42	1.17 ± 0.32	5.14 ± 1.16	F(1,15) = 12.89, p = 0.002, partial ç2 = 0.518	
PANAS Positive Affect	25.00 ± 1.84	16.62 ± 0.97	16.62 ± 0.90	F(1,17) = 19.56, p < 0.001, partial ç2 = 0.620	
PANAS Negative Affect	12.08 ± 0.67	10.92 ± 0.31	18.53 ± 2.61	F(1,13) = 7.45, p = 0.015, partial ç2 = 0.383	
Heart rate (bpm)	74.38 ± 3.03	78.08 ± 3.37	92.15 ± 4.95	F(2,19) = 12.26, p = 0.001, partial ç2 = 0.505	
Systolic blood pressure (mmHg)	122.54 ± 4.72	115.23 ± 3.27	129.31 ± 6.62	F(2,22) = 6.57, p = 0.007, partial ç2 = 0.354	
Diastolic blood pressure (mmHg)	72.46 ± 2.46	70.38 ± 1.92	71.31 ± 2.81	n.s.	
Values are reported as mean ± standard error. Abbreviations: GAD-7, Generalized Anxiety Disorder Screener; PANAS, Positive and Negative Affect Schedule; bpm, beats per minute; mmHg, millimeters of mercury; n.s., not significant.	


Supplementary Table S3: Clusters of significant activation in the passive emotional face perception task, All faces > Control.
		Coordinates of peak voxel (MNI)					
Anatomical Region	x	y	z	Number of voxels	Z value	Corrected p	BA	
Right									
	Middle frontal gyrus extending to OFC	46	16	30	679	9.77	9.18 x 10^-17^	44	
	Fusiform cortex	40	-58	-18	130	9.85	4.23 x 10^-17^	37	
	Amygdala	22	-4	-10	83	8.23	7.08 x 10^-11^	53	
	Precentral gyrus	48	4	50	72	7.03	4.78 x 10^-7^	6	
	Superior temporal gyrus	52	-28	-2	37	7.23	1.22 x 10^-7^	22	
	Antero-inferior temporal lobe	42	-4	-34	35	6.24	6.90 x 10^-5^	20	
	Premotor cortex	8	14	58	24	6.97	7.04 x 10^-7^	6	
	Superior frontal gyrus	14	12	66	20	6.01	0.000263	6	
	Angular gyrus	54	-44	14	18	7.15	2.08 x 10^-7^	39	
	Occipital cortex	52	-74	-2	14	5.95	0.000368	19	
Left									
	Lateral orbitofrontal cortex	-48	32	-8	179	7.01	5.27 x 10^-7^	47	
	Fusiform cortex	-42	-50	-18	140	9.90	2.55 x 10^-17^	37	
	Amygdala	-16	-8	-12	102	8.08	2.24 x 10^-10^	53	
	Precentral gyrus	-40	-2	46	58	6.90	1.14 x 10^-6^	6	
	Supramarginal gyrus	-60	-46	30	46	6.85	1.56 x 10^-6^	39	
	Ventrolateral PFC	-54	18	26	44	5.91	0.000461	44	
	Angular gyrus	-60	-58	14	11	6.60	7.60 x 10^-6^	39	
	Temporo-occipital cortex	64	-58	10	10	6.09	0.000162	37	
Clusters with extent greater than 10 voxels shown. Abbreviations: MNI, Montreal Neurological Institute; BA, Brodmann area; OFC, orbitofrontal cortex; PFC, prefrontal cortex.	


Supplementary Table S4: Correlations between task-related functional connectivity changes in the contrast angry > happy and CO2 challenge outcome measures with moderate-to-large effect size (r > ±0.600)
Brain regions	Outcome measure	Pearson's correlation coefficient (r)	t-statistic	
vmPFC-right amygdala	Heart rate (bpm)	0.831	t(11) = 4.96	
	GAD-7	0.700	t(11) = 3.25	
MCC-left amygdala	GAD-7	-0.726	t(11) = -3.50	
	PANAS Negative Affect	-0.630	t(11) = -2.69	
	Heart rate (bpm)	-0.621	t(11) = -2.63	
	Diastolic blood pressure (mmHg)	-0.604	t(11) = -2.51	
Left supramarginal-right angular gyrus	Heart rate (bpm)	0.637	t(11) = 2.74	
Right thalamus-SMA	PANAS Positive Affect	-0.609	t(11) = -2.54	
SMA-vmPFC	Heart rate (bpm)	0.722	t(11) = 3.46	
	Diastolic blood pressure (mmHg)	0.623	t(11) = 2.64	
SMA-left amygdala	Heart rate (bpm)	0.627	t(11) = 2.67	
Abbreviations: GAD-7, Generalized Anxiety Disorder Screener; PANAS, Positive and Negative Affect Schedule; bpm, beats per minute; mmHg, millimeters of mercury; vmPFC, ventromedial prefrontal cortex; MCC, midcingulate cortex; SMA, supplementary motor area.
